# The biochemical and mass spectrometric profiling of the dystrophin complexome from skeletal muscle

**DOI:** 10.1016/j.csbj.2015.11.002

**Published:** 2015-11-26

**Authors:** Sandra Murphy, Kay Ohlendieck

**Affiliations:** Department of Biology, Maynooth University, National University of Ireland, Maynooth, Co. Kildare, Ireland

**Keywords:** Complexome profiling, Cytoskeleton, Dystrophin-glycoprotein complex, Dystrophinopathy, Extracellular matrix

## Abstract

The development of advanced mass spectrometric methodology has decisively enhanced the analytical capabilities for studies into the composition and dynamics of multi-subunit protein complexes and their associated components. Large-scale complexome profiling is an approach that combines the systematic isolation and enrichment of protein assemblies with sophisticated mass spectrometry-based identification methods. In skeletal muscles, the membrane cytoskeletal protein dystrophin of 427 kDa forms tight interactions with a variety of sarcolemmal, cytosolic and extracellular proteins, which in turn associate with key components of the extracellular matrix and the intracellular cytoskeleton. A major function of this enormous assembly of proteins, including dystroglycans, sarcoglycans, syntrophins, dystrobrevins, sarcospan, laminin and cortical actin, is postulated to stabilize muscle fibres during the physical tensions of continuous excitation-contraction-relaxation cycles. This article reviews the evidence from recent proteomic studies that have focused on the characterization of the dystrophin-glycoprotein complex and its central role in the establishment of the cytoskeleton-sarcolemma-matrisome axis. Proteomic findings suggest a close linkage of the core dystrophin complex with a variety of protein species, including tubulin, vimentin, desmin, annexin, proteoglycans and collagens. Since the almost complete absence of dystrophin is the underlying cause for X-linked muscular dystrophy, a more detailed understanding of the composition, structure and plasticity of the dystrophin complexome may have considerable biomedical implications.

## 1 Introduction

Following the establishment of the mass spectrometry-based draft of the human proteome and its variation in different tissues [1], [2], [3], a new emphasis of proteome-wide studies is the detailed elucidation of genotype-phenotype relationships at the level of interactome networks [4]. The systematic application of target proteomics and the detailed characterization of complexomes promise new insights into proteome-wide alterations due to developmental processes, physiological adaptations, pathological insults or natural aging [5], [6], [7]. A large number of bioinformatics tools are available to assess proteome-wide predictions of protein-protein interaction patterns in health and disease [8], [9], [10], [11], [12]. In skeletal muscle proteomics, comprehensive studies focusing on the systematic cataloguing of the protein constituents of contractile tissues have been carried out over the last decade and established thousands of distinct protein species being present in the most abundant type of tissue in the mammalian body [13], [14], [15], [16], [17], [18]. Building on these proteomic maps, it is now possible to determine the specific arrangement and latent plasticity of protein-protein interaction patterns within large protein complexes from skeletal muscles. Contractile fibres contain considerable numbers of extremely high-molecular-mass proteins and many membrane-associated supramolecular protein complexes. Such protein species are difficult to study using standard biochemical and biophysical techniques. However, the extraordinary advances made in large-scale protein separation methods and the development of highly sensitive mass spectrometers has drastically improved the capabilities of studying very large proteins and multi-subunit complexes [19].

The giant class of muscle proteins is exemplified by titin, nebulin and obscurin, crucial molecular players that provide fibre elasticity, stretch response and sarcomeric organization [20]. Large protein complexes from skeletal muscles are represented by the ryanodine receptor calcium release channel of the triad junction, the dihydropyridine receptor of the transverse tubules and the dystrophin-associated glycoprotein complex of the sarcolemma [21], [22]. This makes skeletal muscle an ideal system for the study of the formation and stabilization of very large protein complexes, as well as determining the potential susceptibility of supramolecular protein structures to proteolysis and degradation under pathophysiological conditions. This review presents an overview of recent proteomic investigations that have focused on the mass spectrometric analysis of dystrophin and its associated glycoprotein complex. Since the results of comparative proteomic studies of muscular dystrophy and the determination of secondary effects downstream of dystrophin deficiency have previously been reviewed [23], [24], [25], these aspects of the proteomic analysis of the dystrophin complex will not be covered in detail. Instead, this article provides a comprehensive account of the mass spectrometric analysis of the dystrophin-associated complexome and its central role in the trans-sarcolemmal linkage between the basement membrane and the intracellular actin cytoskeleton.

## 2 Dystrophin

The positional cloning strategy that was used in the molecular genetic analysis of X-linked muscular dystrophy resulted in the ground-breaking discovery of the dystrophin gene [26]. The *Dmd* gene represents the largest identified gene in the human genome [27]. It contains 79 exons and exhibits a highly complex arrangement of 7 promoters that drive the expression of 3 full-length Dp427 isoforms and 4 shorter isoforms named Dp260, Dp140, Dp116 and Dp71 [28]. The tissue-specific dystrophin species are Dp427-M in striated muscle fibres, Dp427-B in brain, Dp427-P in Purkinje neurons, Dp-260-R isoform in retina, Dp-140-B/K in brain and kidney tissues and Dp-116-S in Schwann cells [29]. The smallest dystrophin isoform Dp-71-G is ubiquitous with high levels in the central nervous system [30].

The molecular structure of the full-length Dp427 protein from skeletal muscle includes a unique carboxy-terminal (CT) domain, a cysteine-rich (CR) domain (including a WW-domain protein-binding motif, a ZZ module and an EF hand Ca^2 +^-dependent region), a central rod domain characterized by 24 spectrin-like repeats (SLR 1-3, SLR 4-19 and SLR 20-24) interspersed by 4 proline-rich hinge regions H1 to H4, and an amino-terminal domain with calponin homology units [31], [32]. The Dp427-M isoform contains the major binding sites for cortical actin in the amino-terminus and the central rod domain, as well as interaction zones for neuronal nitric oxide synthase nNOS, β-dystroglycan, syntrophins and dystrobrevins in the rod domain, the most distal hinge region, the cysteine-rich domain and the carboxy-terminal domain, respectively [33].

Striking similarities of large structural domains within the Dp427 molecule with the prototypical cytoskeletal proteins β-spectrin and α-actinin established the principal role of dystrophin as a stabilizing linker at the sarcolemma-actin interface in striated muscles [34]. Dystrophin of 427 kDa has characteristic biochemical properties of a membrane cytoskeletal protein [35] and is recognized as a major member of the spectrin-type super family of actin-binding proteins that are proposed to have originated from a common ancestral α-actinin molecule [36]. Immuno electron-microscopical studies of normal skeletal muscle localized dystrophin to the fibre periphery on the cytoplasmic face of the sarcolemma [37]. The detailed biochemical and cell biological characterization of the protein product of the *Dmd* gene established that full-length dystrophin does not exist in isolation at the sarcolemma membrane, but forms tight interactions with a variety of other muscle proteins [38].

## 3 Dystrophin-associated proteins

The systematic application of lectin-based methods was of central importance for the detailed biochemical analysis of dystrophin. The plant lectin wheat germ agglutinin is frequently employed for the isolation and characterization of glycoproteins containing N-acetylglucosamine and sialic acid residues [39] and was instrumental for the biochemical enrichment of the dystrophin complex. Since the Dp427 molecule itself is not glycosylated [31], the tight binding of dystrophin to lectin-containing beads was interpreted as indirect interactions via associated glycoproteins [40]. Lectin affinity techniques were used in combination with cell biological, structural and biochemical methods to establish the composition of the dystrophin-glycoprotein complex in both normal and dystrophic muscle fibres [41]. This included the analysis of the dystrophin-glycoprotein complex in affinity-purified sarcolemma vesicles [35], [42] and the related utrophin-glycoprotein complex of the neuromuscular junction [43], the biochemical isolation and characterization of the dystrophin-associated glycoprotein complex from detergent-solubilized microsomal preparations [40], [44], [45], [46] and the purification of skeletal muscle dystrophin to homogeneity [47].

Since the original discovery of the dystrophin-associated glycoprotein complex [44], a large number of studies have characterized the various components attached to dystrophin [48]. Dystrophin-associated proteins can be divided into sarcolemmal proteins (β-dystroglycan, α-sarcoglycan, β-sarcoglycan, γ-sarcoglycan, δ-sarcoglycan, sarcospan), cytosolic proteins (dystrobrevins, syntrophins, nNOS) and extracellular proteins (α-dystroglycan, laminin) [41]. This article focuses on the proteomic identification and characterization of the dystrophin complexome. For detailed descriptions of the initial biochemical and cell biological characterization of the dystrophin-glycoprotein complex prior to its proteomic evaluation, please see extensive reviews [22], [41], [48], [49]. Distinct molecular linkages underlie the coupling between dystrophin and the sarcolemmal glycoprotein β-dystroglycan, dystrophin and cortical actin, and dystrophin and the cytoplasmic components of the dystrophin-glycoprotein complex, including syntrophins and dystrobrevins. Domains within the Dp427 molecule with crucial binding sites are represented by the amino-terminal domain, the central part of the spectrin-like repeats, the cysteine-rich domain and the coiled-coil region at the carboxy terminus.

Besides the core members of the dystrophin-associated glycoprotein complex, various physiological receptors, signalling proteins, cytoskeletal elements and extracellular components are indirectly linked to dystrophin forming a large protein network at the cytoskeleton-sarcolemma-matrisome axis. A major dystrophin-associated signalling enzyme is the neuronal nitric oxide synthase nNOS that is involved in the regulation of skeletal muscle function and metabolism [50]. This crucial enzyme is linked to both α1-syntrophin and dystrophin [51]. Its enzymatic product, the second messenger nitric oxide, functions in key signal transduction pathways that modulate oxidative metabolism, carbohydrate metabolism, vasodilatation, neuromuscular transmission and fibre contractility [52]. Voltage-gated Na^+^-channels were shown to be linked via syntrophins to the dystrophin-glycoprotein complex, which in turn anchors these ion channels indirectly to the actin membrane cytoskeleton and the basal lamina [53]. Tight interactions also exist intracellularly between plectin and dystrophin and its associated glycoproteins. Plectin of approximately 530 kDa is a key linker molecule that maintains interactions within the cytoskeletal network consisting of actin filaments, microtubules and intermediate filaments. It was shown to tether desmin-containing intermediate filaments to dystrophin and vinculin, thus playing a central role in the stabilization of the subsarcolemmal region during excitation-contraction-relaxation cycles [54], [55], [56].

On the extracellular site, the dystrophin complex interacts with the small leucine-rich repeat proteoglycan biglycan of the matrisome. Biglycan appears to regulate the expression and localization of a variety of dystrophin-associated proteins, including sarcoglycans, dystrobrevins, syntrophins and nNOS [57], [58], [59]. In analogy to dystrophin, the dystrophin-related protein utrophin, which is located mostly at the neuromuscular junction [60], also interacts with dystrophin-associated glycoproteins [61]. A key utrophin-associated protein is represented by α-dystroglycan, which functions as an extracellular receptor for the large proteoglycan agrin at the postsynaptic membrane of the neuromuscular junction [62], [63], [64]. Since agrin is essential for the development and specialization of the neuromuscular junction and aggregation of the nicotinic acetylcholine receptor complex during synaptogenesis [65], the utrophin/dystrophin-glycoprotein complex at the sarcolemma/neuromuscular junction plays an essential role in neurotransmission [66].

**Fig. 1:**
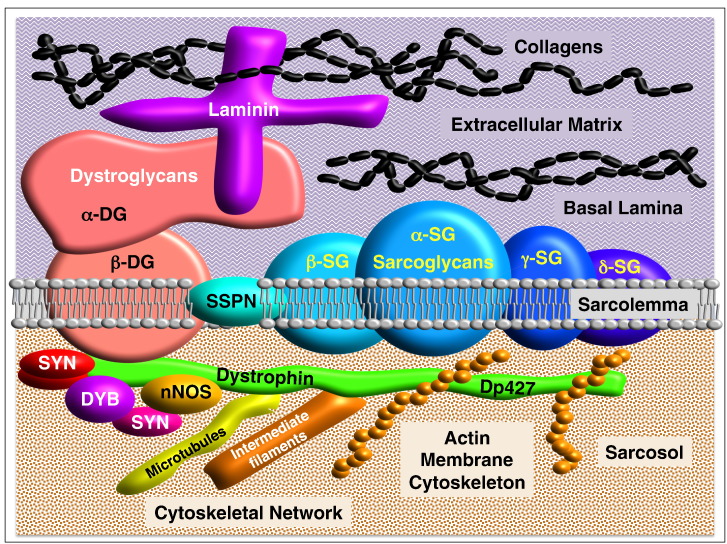
Organization of the dystrophin-glycoprotein complex. The dystrophin-glycoprotein complex is a core element of the extracellular matrix-sarcolemma-cytoskeleton axis and provides stability to the surface membrane structure of skeletal muscle fibres during excitation-contraction-relaxation cycles. The main sarcolemmal complex consists of the extracellular proteins α-dystroglycan (α-DG) and laminin, the plasmalemmal proteins β-dystroglycan (β-DG), α-sarcoglycan (α-SG), β-sarcoglycan (β-SG), γ-sarcoglycan (γ-SG), δ-sarcoglycan (δ-SG) and sarcospan (SSPN), and the cytosolic proteins dystrophin (Dp427), syntrophins (SYN), dystrobrevins (DYB), cortical actin and the enzyme neuronal nitric oxide synthase nNOS. Collagen molecules are linked to the dystrophin-glycoprotein complex through interactions with laminin in the extracellular matrix, and the actin membrane cytoskeleton forms together with microtubules and intermediate filaments the intracellular matrix of muscle fibres.

The arrangement of the core members of the dystrophin-glycoprotein complex is illustrated in Fig. 1, including the interactions between (i) collagen, laminin and α-dystroglycan in the extracellular matrix, (ii) β-dystroglycan, α-β-γ-δ-sarcoglycans and sarcospan within the sarcolemma and (iii) dystrophin, syntrophins, dystrobrevins, nNOS, cortical actin, tubulin and intermediate filament components in the sarcosol. This large sarcolemma-bridging protein network provides crucial input into surface membrane stability, signal transduction and plasmalemmal receptor anchoring. This is exemplified by the fact that deficiency in the membrane cytoskeletal protein dystrophin, due to primary abnormalities in the *Dmd* gene [26], triggers the highly progressive muscle wasting disease named Duchenne muscular dystrophy [67]. The almost complete lack of dystrophin results in the drastic reduction of dystrophin-associated glycoproteins [68], [69], [70], illustrating the complexity and interconnectivity of the dystrophin network in skeletal muscle fibres.

## 4 Proteomics of the dystrophin complexome

### 4.1 Proteomic profiling of dystrophin

Since alterations in dystrophin expression are primarily involved in Duchenne muscular dystrophy, Becker muscular dystrophy and X-linked dilated cardiomyopathy [71], [72], [73], mass spectrometry-based proteomics has been mostly applied in comparative biomedical studies [23]. The initial proteomic profiling of muscle biopsies and biofluids has almost exclusively resulted in the identification of secondary alterations in the muscle proteome downstream of the dystrophin-glycoprotein complex, as reviewed by Dowling et al. [25]. This is probably due to the fact that the full-length Dp427-M isoform and its associated protein complex are difficult to identify and characterize by standard proteomics using whole tissue extracts. Skeletal muscle proteins that: (i) are of relatively low abundance, (ii) have a large molecular mass, (iii) exist in tight association with a variety of other protein species and (iv) are directly or indirectly linked to the hydrophobic environment of biomembranes often present technical challenges for routine biochemical and mass spectrometric studies [19], [74]. However, the application of elaborate pre-fractionation steps, extensive purification approaches and advanced mass spectrometric analyses have more recently resulted in the proteomic identification and characterization of dystrophin and the dystrophin-associated complex [75], [76], [77], [78], [79], [80], [81], [82].

### 4.2 Mass spectrometric characterization of the purified dystrophin complex

Subproteomic studies that focus on systematic cataloguing or the comparative analysis of isolated organelles or distinct subcellular fractions have the marked advantage of dealing with reduced sample complexity [83]. This usually enables the swift identification of a wider range of protein species [84]. The usage of subcellular fractionation and protein enrichment strategies on the one hand, and the combination of gel electrophoresis and liquid chromatography for the optimum separation of diverse protein populations on the other hand, allows detailed insights into the otherwise hidden constellation of low copy number proteins from skeletal muscles [85]. For the subproteomic analysis of dystrophin and its associated glycoprotein complex in highly enriched sarcolemma vesicles, an elaborate pre-fractionation protocol was applied [75]. This included differential centrifugation steps for the isolation of crude surface membranes followed by density gradient ultracentrifugation to harvest enriched membrane fractions. To remove cross-contaminating subcellular structures derived from transverse tubules and the highly abundant sarcoplasmic reticulum, lectin affinity agglutination and mild detergent washing was employed to isolate sarcolemma vesicles. Since standard two-dimensional gel electrophoresis and in-gel digestion had failed to identify the dystrophin-glycoprotein complex during early proteomic studies of muscular dystrophy [23], [25], a combination of one-dimensional gradient gel electrophoresis, electrophoretic protein transfer and on-membrane digestion were applied prior to mass spectrometric analysis [86].

Certain technical issues when studying low-abundance and high-molecular-mass proteins, such as insufficient protein sequence coverage due to poor digestion by standardized in-gel methodology, can be overcome with on-membrane digestion following gradient gel electrophoresis [87]. The proteomic analysis of sarcolemma vesicles from skeletal muscle identified the plasmalemma marker dysferlin and key members of the dystrophin-glycoprotein complex, including the Dp427-M isoform of full-length dystrophin, α-sarcoglycan and α-syntrophin [75]. Following the successful identification of sarcolemmal dystrophin by mass spectrometry, a similar on-membrane digestion approach was used to study the purified dystrophin complex. The core assembly of dystrophin and its tightly associated glycoproteins was solubilized by mild digitonin treatment of microsomes and then isolated by wheat germ agglutinin chromatography, ion exchange chromatography and a final density gradient ultracentrifugation step. One-dimensional gel electrophoresis in combination with on-membrane digestion and mass spectrometry resulted in the identification of dystrophin isoform Dp427-M and its associated protein dystrobrevin, as well as the biochemical documentation of the integral proteins α-sarcoglycan and γ-sarcoglycan [75]. Mass spectrometric methods were also used to characterize a panel of tryptic peptides from the dystrophin molecule [88] and for the quantitation of muscle dystrophin by combining stable isotope labelled Dp427 as a spike-in standard [76].

### 4.3 Proteomic analysis of the immunoprecipitated dystrophin complex

Co-immunoprecipitation is a convenient method for the enrichment of multi-subunit protein complexes and their associated components [89]. Ideally, a highly sensitive and specific antibody is used in affinity purification-mass spectrometry for studying protein interactomes. In relation to the dystrophin complex, immunoprecipitation was used by Yoon et al. [78] for the comparative proteomic profiling of dystroglycan-associated proteins in wild type, *mdx* and *Galgt2* transgenic mouse skeletal muscle. The mass spectrometric analysis of precipitated proteins using a monoclonal antibody to β-dystroglycan, which had been bound and cross-linked to protein G-conjugated magnetized beads, resulted in the identification of full-length dystrophin isoform Dp427, full-length utrophin isoform Up395, α-dystroglycan, β-dystroglycan, α-sarcoglycan, β-sarcoglycan, γ-sarcoglycan, δ-sarcoglycan, α1-dystrobrevin, α2-dystrobrevin, α1-syntrophin, β1-syntrophin and β2-syntrophin. Indirect binding partners belonging to the dystrophin complexome were established to include proteins associated with the contractile apparatus, including fast troponin TnT3, slow troponin TnT1, α-actinin-3, myozenin 1 and nebulin. Besides dystrophin, all these regulatory and stabilizing elements of the acto-myosin complex were shown to be greatly reduced in skeletal muscle extracts from the *mdx* mouse model of X-linked muscular dystrophy [78]. In relation to muscular dystrophy, important findings of this immunoprecipitation/mass spectrometry study were the demonstration that the dystroglycan subcomplex exhibits a different protein interaction profile in *mdx* muscles and that overexpression of the synaptic muscle glycosyltransferase Galgt2 can modify α-dystroglycan and thereby counteract dystrophic symptoms [78].

A comparative immunoprecipitation study using an antibody to dystrophin showed tissue-related differences in the association of Dp427 with syntrophins, nNOS and dystrobrevins in the heart versus skeletal muscles [77]. The precipitated skeletal muscle complex contained dystrophin isoform Dp427-M, the α/β-dystroglycan subcomplex, the α-β-γ-δ-sarcoglycan subcomplex, α1-β1-syntrophins, sarcospan, α-dystrobrevin and nNOS. Novel Dp427-associated proteins in cardiac muscle were identified by mass spectrometry as the molecular chaperone αB-crystalline, as well as the caveolae protein Cavin-1, the scaffold protein Ahnak1 and the Z-disc assembly protein Cypher. This suggests that the dystrophin complexome is involved in dissimilar pathways and cellular mechanisms in heart versus muscle fibres. Immunoprecipitation was also employed for studying dystrophin phosphorylation and it was demonstrated that the enhanced phosphorylation of segments within the cysteine-rich domain of Dp427 results in an enhanced association between dystrophin and β-dystroglycan [79].

### 4.4 Identification of dystrophin using comparative tissue proteomics

Although the core members of the dystrophin-glycoprotein complex could be clearly identified by mass spectrometry following immunoprecipitation or enrichment by ion exchange and lectin chromatography [75], [77], [78], the majority of the initial proteomic screenings of dystrophic muscle specimens failed to identify full-length dystrophin [23]. However, recent comparative studies using the microsomal fraction or whole tissue preparations were able to characterize the dystrophin complex and its associated components by proteomic means using more sensitive mass spectrometric methods [80], [81], [82]. Table 1 lists the mass spectrometric identification of core members of the dystrophin complex in microsomal preparations [80]. The protein sarcospan, which was also not properly recognized in the above-described study using immunoprecipitation with an antibody to β-dystroglycan [78], was only identified by 1 peptide. This inefficient coverage during routine proteomic screens could be due to the small size, low abundance and highly hydrophobic nature of sarcospan. The subproteomic survey of the crude microsomal fraction from wild type versus dystrophic *mdx-4cv* hind limb muscles by label-free mass spectrometry established pathological expression changes for 281 proteins [80]. The usage of differential centrifugation effectively reduced sample complexity and the introduction of this pre-fractionation step enabled the simultaneous proteomic analysis of the dystrophin-glycoprotein complex and secondary changes in other muscle components including the wider dystrophin complexome using a single analytical run. Table 2 lists key cytoskeletal proteins that have been identified by LC-MS/MS analysis of the muscle microsomal fraction [80], including vimentin, desmin, vinculin, plectin and tubulin.

Importantly, the proteomic evaluation of the crude membrane fraction from dystrophic skeletal muscle identified the full-length dystrophin isoform Dp427-M as the most significantly reduced protein [80]. Besides drastic reductions in α/β-dystroglycans, δ-sarcoglycan, γ-sarcoglycan and α1-syntrophin, downstream alterations were established for proteins involved in muscle metabolism, cellular signalling, the excitation-contraction-relaxation cycle, ion-handling, protein folding, the cytoskeletal network and the extracellular matrix. A considerable reduction in muscle-associated biomarker candidates was confirmed for the fast isoform of myosin-binding protein MBP-C, myomesin, myozenin isoform MYZ-1, carbonic anhydrase isoform CA3 and the oxygen-transporter myoglobin [80]. The most significantly increased protein species in microsomes from *mdx4cv* muscles was shown to be α1-antitrypsin, a multi-functional protein with major anti-protease and anti-inflammatory properties. The up-regulation of antitrypsin might therefore be a protective mechanism in muscular dystrophy. The concept of a dystrophin complexome with linkages to the cytoskeletal network was confirmed by the mass spectrometric identification of substantial increases in the microtubular component tubulin, vinculin and the intermediate filament protein vimentin [80]. Alterations in annexins were shown to occur in relation to isoforms AnxA1, AnxA2, AnxA4, AnxA5 and AnxA6 and agree with pathophysiological disturbances in Ca^2 +^-buffering and membrane organization [90]. A novel proteomic biomarker of muscular dystrophy might be represented by the ouabain-sensitive Na^+^/K^+^-ATPase, which was found to be increased in the Dp427-deficient skeletal muscles [80]. The higher density of this electrogenic ion pump is probably involved in the physiological stabilisation of the membrane potential in damaged skeletal muscle fibres and may thereby compensate abnormal Na^+^- and K^+^-ion fluxes over the Dp427-deficient sarcolemma membrane [91].

The general perturbation of the extracellular matrix and the cytoskeletal network in dystrophic fibres was confirmed by proteomic analyses of whole tissue preparations [81], [82], [92]. The fact that the Dp427-M isoform of dystrophin was identified as the most significant change in total muscle extracts from the *mdx4cv* model of dystrophinopathy perfectly agrees with the idea that the deficiency in dystrophin is the primary abnormality in Duchenne muscular dystrophy. The manifestation of serious side effects in dystrophic patients due to myofibrosis [93] was substantiated by the identification of increased levels of various isoforms of collagen and associated markers of the extracellular matrix, including asporin, decorin, fibronectin, prolargin, mimecan, biglycan and lumican [92]. The large number of altered proteins belonging to the dystrophin complexome supports the findings from previous comparative proteomics studies [94], [95], [96], [97], [98], [99] and suggests a number of robust and universal biomarkers of muscular dystrophy [100]. A panel of these protein markers might be useful for designing improved predictive, diagnostic, prognostic and therapy-monitoring assay systems in the future [101].

**Fig. 2:**
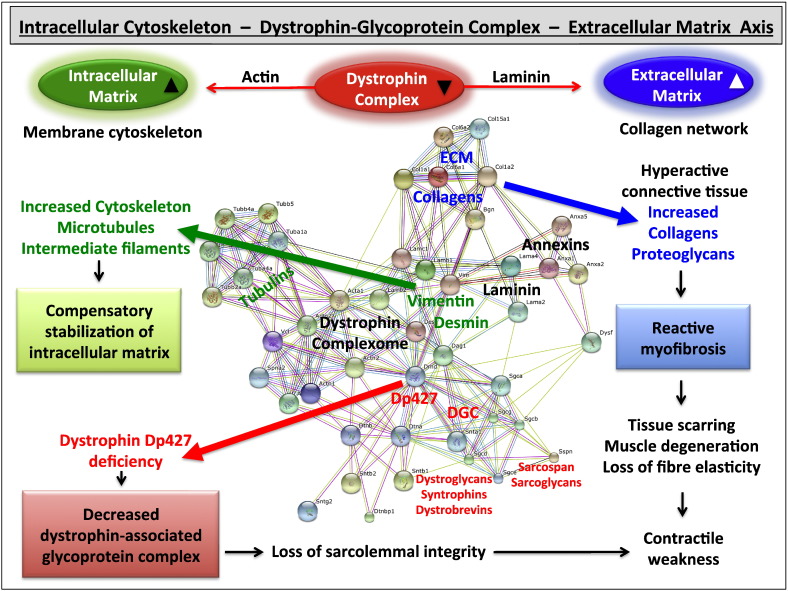
Overview of the dystrophin complexome. The diagram shows the interaction patterns between collagens, tubulins, annexins, vimentin, desmin and the dystrophin-glycoprotein complex as part of the intracellular matrix-sarcolemma-extracellular matrix axis from mature skeletal muscle. The central panel depicts an overview of protein-protein interactions, based on analysis using the bioinformatics database STRING [102]. Detailed protein networks related to the dystrophin complexome and its pathobiochemical changes in X-linked muscular dystrophy have been recently published [80], [81]. The flowcharts surrounding the proteomic map highlight major changes in Dp427-deficient muscle fibres, including a compensatory up-regulation of microtubules and intermediate filaments, a loss of sarcolemmal integrity due to the loss of the dystrophin complex, and reactive myofibrosis caused by a hyperactive connective tissue.

The above-described findings from systematic proteomic studies indicate that dystrophin interacts tightly with the core members of the dystrophin-associated glycoprotein complex, such as dystroglycans, sarcoglycans, syntrophins, dystrobrevins and sarcospan, but also forms indirect linkages with a large variety of other protein species, including tubulin, vimentin, desmin, annexin and collagens. The bioinformatics STRING analysis [102] shown in Fig. 2 summarizes the concept of a dystrophin complexome. More detailed proteomic maps of the dystrophin-associated protein network and proposed cytoskeleton-sarcolemma-matrisome axis have recently been published [80], [81].

## 5 Summary and outlook

The precise determination of the localization of protein isoforms in multiple subcellular compartments and the elucidation of the dynamic nature of protein interactions are of crucial importance for understanding cell biological processes at the level of the proteome. Building on systems biological knowledge, it will be easier to comprehend physiological adaptations and pathophysiological changes in highly intricate cellular assemblies. Both the reduction of sample complexity prior to the affinity purification of the target protein complex and the application of advanced mass spectrometric methods promises to improve the characterization of supramolecular protein complexes in heterogeneous and highly adaptable tissues, such as skeletal muscles. Over the last few years, new proteomic approaches have helped to better identify direct and indirect binding partners of the membrane cytoskeletal protein dystrophin. Besides the already previously established tight interactions with various cytosolic, sarcolemmal and extracellular proteins, many core elements of the collagen-associated extracellular matrix and the intracellular cytoskeletal network have been shown to be linked to the dystrophin-glycoprotein complex. The sarcolemmal assembly of dystrophin, utrophin, dystroglycans, sarcoglycans, syntrophins, dystrobrevins and sarcospan mediates cellular signalling events and anchors critical surface proteins to the underlying membrane cytoskeleton. Most importantly, the provision of an indirect linkage between laminin at the outside of the muscle fibres and cortical actin at the inside of the sarcolemma via a dystrophin lattice stabilizes the extracellular matrix-plasmalemma-cytoskeleton axis.

Comparative proteomic studies have clearly established that this large interconnection between muscle proteins is disturbed in Duchenne muscular dystrophy. The deficiency in dystrophin appears to trigger major damage to sarcolemmal integrity, causes increased myofibrosis and results in the compensatory up-regulation of microtubules and intermediate filaments. Future proteomic studies of protein-protein interactions within the wider dystrophin complex will hopefully shed more light on these disease-related rearrangements in the dystrophin complexome. This might include the application of the TAP (tandem affinity purification) tagging method or sophisticated XL-MS (chemical cross-linking mass spectrometry) techniques. Studying the dystrophin complex using targeted proteomics and systems biological approaches promises new insights into the molecular pathogenesis of X-linked muscular dystrophy and might therefore make an important contribution to translational medicine and skeletal muscle pathology.

## 6 Competing interests

The authors have declared that no competing interests exist.

## Figures and Tables

**Table 1:** Mass spectrometry-based identification of dystrophin and closely associated proteins. The proteomic profiling of the dystrophin-glycoprotein complex and associated proteins was carried out by LC-MS/MS analysis using the microsomal fraction from mouse skeletal muscle [80].

Accession No.	Protein name	Coverage (%)	Unique peptides	Molecular mass (kDa)
P11531	Dystrophin Dp427-M	10.85	26	425.6
Q62165	Dystroglycan	10.41	9	96.8
P82350	Sarcoglycan, alpha	19.12	6	43.3
P82349	Sarcoglycan, beta	29.06	5	34.9
P82347	Sarcoglycan, delta	18.34	4	32.1
P82348	Sarcoglycan, gamma	14.78	4	32.1
Q62147	Sarcospan	4.17	1	23.8
Q9D2N4	Dystrobrevin, alpha	5.63	3	84.0
Q61234	Syntrophin, alpha-1	7.95	3	53.6
Q60675	Laminin, alpha-2	7.60	13	343.6
P02469	Laminin, beta-1	2.13	3	197.0
Q61292	Laminin, beta-2	2.33	3	196.5
P02468	Laminin, gamma-1	9.15	10	177.2

**Table 2:** Mass spectrometry-based identification of key cytoskeletal proteins from skeletal muscle. The proteomic profiling of cytoskeletal proteins was carried out by LC-MS/MS analysis using the microsomal fraction from mouse skeletal muscle [80].

Accession No.	Protein name	Coverage (%)	Unique peptides	Molecular mass (kDa)
P20152	Vimentin	46.14	15	53.7
P31001	Desmin	71.64	37	53.5
Q64727	Vinculin	18.39	13	116.6
Q9QXS1	Plectin	19.95	68	533.9
P68369	Tubulin, alpha-1A	53.44	7	50.1
P68368	Tubulin, alpha-4A	56.70	7	49.9
Q9JJZ2	Tubulin, alpha-8	36.30	4	50.0
Q7TMM9	Tubulin, beta-2A	47.42	1	49.9
P68372	Tubulin, beta-4B	66.74	3	49.8
P99024	Tubulin, beta-5	66.89	5	49.6
Q922F4	Tubulin, beta-6	21.92	1	50.1
